# Multimodal Exercise and Nutritional Interventions in Pediatric Cancer: Effects on Physical Function, Body Composition, and Metabolic Health—A Narrative Review

**DOI:** 10.3390/children13060729

**Published:** 2026-05-24

**Authors:** Antonio Ibáñez-Camacho, Belén Pastor-Villaescusa, Jose Manuel Jurado-Castro, Mercedes Gil-Campos, Francisco Jesus Llorente-Cantarero

**Affiliations:** 1 Maimonides Biomedical Research Institute of Cordoba (IMIBIC), Reina Sofia University Hospital, University of Cordoba, 14071 Cordoba, Spain; antonioibacam@gmail.com (A.I.-C.); belen.pastor@imibic.org (B.P.-V.); mercedes_gil_campos@yahoo.es (M.G.-C.); m12llcaf@uco.es (F.J.L.-C.); 2 CIBER Fisiopatología de la Obesidad y Nutrición (CIBEROBN), Instituto de Salud Carlos III, 28029 Madrid, Spain; 3 Ciencias de la Actividad Física y el Deporte, Escuela Universitaria de Osuna (Centro Adscrito a la Universidad de Sevilla), 41640 Osuna, Spain

**Keywords:** pediatric leukemia, cancer survivors, exercise, resistance training, body composition, sarcopenic obesity, nutrition therapy, autonomic dysfunction, sleep disturbances

## Abstract

**Highlights:**

**What are the main findings?**

- Pediatric cancer treatment is associated with loss of muscle mass and bone density, increased body fat, and higher long-term metabolic risk.
- Early supervised exercise is both safe and effective for preserving physical function, lean mass, and cardiorespiratory fitness during treatment and survivorship.
- Nutritional care should focus on body composition, adequate protein intake, and micronutrient status, while considering that autonomic dysfunction influences the regulation of metabolism and appetite.

**What are the implications of the main findings?**

- Multimodal exercise and nutritional interventions in pediatric oncology should begin early and combine nutrition, exercise, and regular monitoring rather than focusing only on body weight.
- Future studies should use standardized protocols, objective intensity monitoring, harmonized outcome measures, and longer follow-up to define more precise and clinically useful multimodal exercise and nutritional interventions for children with cancer.

**Abstract:**

Survival rates in pediatric cancer have increased substantially over recent decades. However, children and survivors frequently experience treatment-related alterations in physical function, body composition, bone health, and metabolic regulation. Chemotherapy, glucocorticoid exposure, physical inactivity, nutritional imbalance, and inflammatory and neuroendocrine disturbances may contribute to reduced lean mass, decreased bone mineral density, sarcopenic obesity, and long-term cardiometabolic risk. This narrative review critically summarizes current evidence on multimodal exercise and nutritional interventions in pediatric oncology, with particular attention to their effects on physical function, body composition, nutritional status, and metabolic health. Literature searches were conducted in PubMed, Scopus, and Web of Science up to April 2026, combining contextual evidence with studies evaluating combined exercise and nutritional strategies. Current evidence suggests that structured and supervised exercise, particularly resistance and combined aerobic–resistance training, is feasible and safe, and may improve cardiorespiratory fitness, muscle strength, functional capacity, and body composition. Nutritional care should be individualized, prioritizing adequate protein intake, micronutrient status, periodic reassessment of energy requirements, and body composition rather than relying on BMI alone. Nevertheless, available findings remain limited by small sample sizes, heterogeneous protocols, variable supervision, inconsistent outcome assessment, and limited long-term follow-up. Integrating exercise, nutrition, and regular monitoring into pediatric oncology care may help mitigate treatment-related functional and metabolic complications. Future studies should prioritize adequately powered randomized trials, standardized intervention protocols, objective monitoring of exercise intensity, harmonized body composition and functional outcomes, and longer follow-up to define clinically applicable multimodal care models.

## 1. Introduction

Childhood cancer is a relatively rare disease, but it represents a significant global health burden [1]. Leukemia remains one of the most common and clinically significant malignancies in the pediatric population worldwide. Acute lymphoblastic leukemia (ALL) is the predominant subtype and accounts for approximately 68–70% of pediatric cases. Acute myeloid leukemia (AML) is the second most frequent subtype, representing around 22–25% [2]. Advances in early diagnosis, risk stratification, and therapeutic protocols have substantially improved survival. In countries with a very high Human Development Index, five-year survival rates for ALL now exceed 90% [3].

Treatment of ALL is based on multi-agent systemic chemotherapy and is adjusted according to risk factors such as age, Philadelphia chromosome status, disease subtype, and overall health condition [4]. Therapeutic regimens typically include anthracyclines, vincristine, cyclophosphamide, cytarabine, pegaspargase, 6-mercaptopurine, thioguanine, and glucocorticoids. Concretely, glucocorticoids play a central role due to their capacity to induce apoptosis in lymphoblasts [4]. In selected high-risk or relapsed cases, hematopoietic stem cell transplantation may also be indicated [3].

Despite these improvements in survival, pediatric cancer patients frequently experience numerous short- and long-term adverse effects during active treatment and survivorship. These complications lead to a progressive decline in physical function and increased vulnerability, ultimately increasing the risk of late morbidity and premature mortality [5,6].

**Figure 1:**
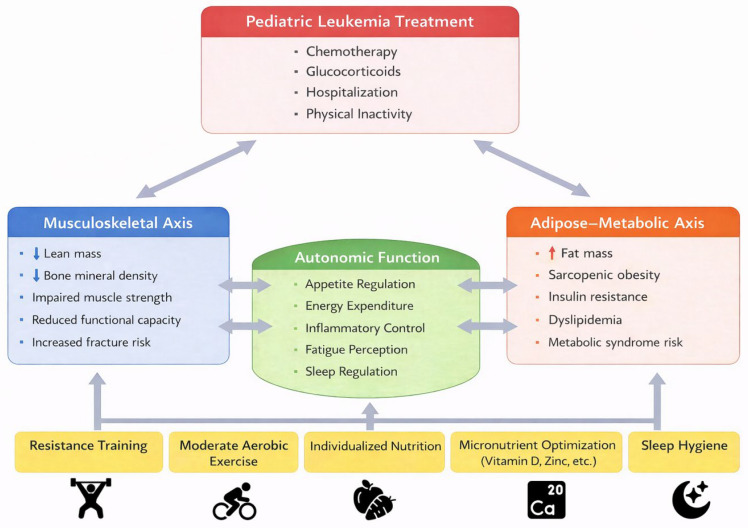
Conceptual multimodal intervention model in pediatric cancer. ↑ indicates an increase; ↓ indicates a decrease.

Novel, multidimensional lifestyle interventions may inform and strengthen clinical practice guidelines to mitigate treatment-related adverse effects. Therefore, this review aims to critically examine how lifestyle interventions may modulate musculoskeletal and adipose metabolic alterations associated with pediatric cancer. Figure 1 summarizes the proposed conceptual framework linking these systems and their modulation by lifestyle interventions.

## 2. Methods

This narrative review was based on three complementary literature search strategies conducted in PubMed, Scopus, and Web of Science databases up to April 2026.

First, a complementary search was conducted to provide contextual, clinical, and mechanistic background on pediatric cancer, including the effects of the disease and its treatment, as well as the role of exercise and nutrition in this population. This search included terms such as “pediatric cancer”, “chemotherapy”, “physical activity”, “exercise”, “nutrition”, “diet”, “cachexia”, “anorexia”, “inflammation”, “GLP-1”, “energy balance”, and “autonomic nervous system”. These studies were not subject to the same inclusion criteria, as their purpose was to support the conceptual framework and interpretation of findings rather than to contribute to the comparative analysis.

Second, a structured search was carried out to identify studies evaluating multimodal lifestyle interventions, specifically combining exercise and nutritional strategies in pediatric oncology. Using Boolean operators, the search strategy combined keywords related to pediatric cancer interventions (e.g., “pediatric”, “child*”, “adolescent*”, “cancer”, “exercise”, “physical activity”, “nutrition”, “diet”, and “lifestyle”). Only studies assessing combined exercise and nutritional interventions, and reporting outcomes related to physical function, body composition, nutritional status or metabolic health, were considered for comparative analysis. Reference lists of relevant reviews were also screened to identify additional studies.

Third, an additional search was conducted to identify studies addressing assessment and monitoring techniques relevant to pediatric oncology, including evaluation of physical function, body composition, and activity levels. These studies were used to contextualize outcome assessment and monitoring approaches within the reviewed interventions.

## 3. Mechanistic Pathways

### 3.1. Musculoskeletal Axis: Loss of Lean Mass and Bone Mineral Density

Exogenous glucocorticoid administration disrupts bone metabolism and is associated with deficits in bone mineral density (BMD) [7,8]. It also induces adverse changes in body composition, including skeletal muscle atrophy and fat redistribution [9]. Several studies have reported significant reductions in BMD among children with ALL following completion of chemotherapy [10,11,12]. Importantly, reduced BMD has also been documented at diagnosis, before therapeutic intervention, suggesting pre-existing skeletal vulnerability [8,13].

In parallel, treatment of pediatric ALL is associated with significant skeletal muscle loss, with an early decline and incomplete recovery pattern [14,15,16]. This effect is partly driven by the action of glucocorticoids on skeletal muscle, promoting myofibrillary atrophy through increased degradation of myosin heavy chains and reduced myosin synthesis. Other factors, such as inadequate nutritional intake and prolonged inactivity, may also contribute to the observed declines in skeletal muscle mass [14].

Beyond direct effects on skeletal muscle, treatment-related factors may also contribute to functional decline at the neuromuscular level. In particular, peripheral neuropathy induced by vincristine can affect nerve conduction and neural signaling. Clinically, this manifests as motor weakness, reduced reflexes, and gait abnormalities, reflecting compromised neuromuscular function [17].

Muscle mass reduction has been positively correlated with “burden of illness”, assessed by total hospitalization days. This suggests that muscle deterioration reflects not only structural changes but also treatment severity and overall morbidity [14].

### 3.2. Adipose–Metabolic Axis: Sarcopenic Obesity and Cardiometabolic Risk

Normal body mass index (BMI) does not necessarily indicate a healthy nutritional status. In cancer, frequently, an increase in fat mass may coexist with reduced lean mass. This pattern, described as sarcopenic obesity, is particularly evident among ALL survivors, in whom adiposity remains elevated while fat-free mass is frequently reduced [18,19,20,21]. Consistent with this phenotype, one cohort study reported sarcopenic obesity in approximately 34% of 65 ALL survivors [18].

Moreover, multiple studies have reported an increased prevalence of overweight and obesity at treatment completion and at five-year follow-up, highlighting the burden of excess adiposity among childhood ALL survivors [22,23]. So, even pediatric cancer survivors can present an elevated cardiometabolic risk profile. In a cohort of 650 adults, long-term survivors of pediatric ALL, the prevalence of metabolic syndrome was 6.9%. Low high-density lipoprotein cholesterol (HDLc), elevated triglycerides, impaired fasting glucose, hypertension, and increased waist circumference were observed in 5.8% to 36.7% of participants, based on the specific metabolic component assessed [24].

### 3.3. Neuroendocrine Mechanisms

Cancer-related anorexia results from a complex interaction between inflammatory, hormonal, and neuroregulatory mechanisms that disrupt hypothalamic control of energy balance [25]. Pro-inflammatory cytokines, such as tumor necrosis factor alpha (TNF-α), interleukin-1 (IL-1), and interleukin-6, suppress appetite and increase energy expenditure [25], while alterations in gut-derived hormones (Glucagon-like peptide-1, peptide YY, ghrelin) further dysregulate hunger-satiety signaling [25,26,27]. In pediatric ALL, higher peptide YY and reduced ghrelin at diagnosis have been associated with changes in BMI and disease burden, supporting their role in anorexia–cachexia development [27]. Reduced heart rate variability during and after leukemia treatment has also been demonstrated, indicating the presence of autonomic dysfunction [28]. Given the established role of parasympathetic activity in regulating gastrointestinal motility, insulin sensitivity, and inflammatory responses [29,30], autonomic imbalance may contribute not only to fatigue and sleep disturbances but also to altered metabolic regulation.

In this context, appetite stimulants such as megestrol acetate or cyproheptadine have been shown to improve weight status in pediatric cancer patients with anorexia or cachexia [31], supporting their clinical use to counteract disease-related weight loss. Considering the impact of anorexia on nutritional status, dietary strategies are a central component of its management, including small, frequent meals, energy-dense foods, and attention to food presentation to improve food intake [25]. Furthermore, PA and sleep optimization have been shown to positively influence autonomic balance [32,33], which suggests a potential therapeutic pathway. In fact, despite interleukin-6 is commonly acknowledged as a pro-inflammatory cytokine, its secretion from skeletal muscle during PA induces anti-inflammatory responses by promoting the synthesis of anti-inflammatory agents like interleukin-10 and IL-1 receptor antagonist, along with the suppression of TNF-α [34].

Overall, cancer-related anorexia should be addressed through an integrated approach combining nutritional support with interventions targeting inflammation and autonomic regulation, reinforcing the rationale for multimodal lifestyle strategies in pediatric cancer.

## 4. Modulatory Interventions

### 4.1. Personalized Nutrition

Children with cancer face a higher risk of malnutrition and cachexia because of multiple factors, including low PA, increased energy needs from growth, disease-related inflammation, and treatment side effects such as muscle protein catabolism and insulin-resistance [25,35]. Severe cases, particularly during stem cell transplantation, exacerbate these issues with symptoms like diarrhea and nausea [36]. For this reason, it is important to customize the nutritional support according to needs, ensuring an adequate energy balance and macro and micronutrient intake, paying special attention to protein intake. It is essential to preserve lean muscle mass due to the greater catabolism that frequently appears in these patients [16].

Several studies have documented suboptimal dietary patterns among pediatric cancer survivors. Overall, caloric intake is usually lower than the recommended daily intake, with insufficient consumption of fruits, vegetables, fiber, dairy products, and water [37]. Zhang et al. (2015) [38] reported poor adherence to dietary recommendations, including low intake of plant proteins, seafood, potassium, and fiber, with saturated fat consumption exceeding the recommendations. Specifically, this study reported no differences in diet quality between normal-weight and overweight survivors, highlighting that BMI alone may fail to capture underlying nutritional risk [38]. On the other hand, evidence reported in a recent systematic review indicates that resting energy expenditure (REE) in pediatric oncology patients may not be static, showing an increase at diagnosis and a decreased REE during or after treatment [39].

Other options to improve nutritional status according to specific needs involve designing specific diet models. Ketogenic diet (KD) has been explored as a complementary metabolic therapy in oncology due to its theoretical capacity to limit glucose availability to glycolysis-dependent tumor cells [40]. In pediatric populations, evidence is largely restricted to small observational studies in children with brain tumors, primarily assessing feasibility and safety rather than antitumor efficacy [41].

Currently, there are no robust clinical trials evaluating KD in children with leukemia. Limited pilot data in adults suggest that ketosis may be compatible with chemotherapy and could modulate cellular metabolic markers [42]. Nevertheless, these preliminary findings cannot be extrapolated to pediatric patients with leukemia, given the distinct physiological, developmental, and treatment-related contexts.

Alterations in micronutrient status are highly prevalent in pediatric oncology. In a small cohort of children with cancer, almost all patients presented at least one micronutrient deficiency, most commonly affecting vitamin D, vitamin C, and zinc, with prevalence rates markedly higher than those observed in the general pediatric population. Importantly, these deficiencies were not consistently explained by dietary intake and were associated with clinical symptoms such as nausea and infection risk [37]. Similarly, dietary assessments in survivors indicate poor adherence to nutritional recommendations, particularly for potassium, calcium, and vitamin D, together with excessive sodium intake [38]. Consistent with these findings, observational data have demonstrated progressive reductions in serum 25-hydroxyvitamin D levels over time among pediatric cancer survivors [43].

Hence, vitamin D deserves particular attention due to its central role in bone mineralization, calcium homeostasis, and skeletal integrity. Its deficiency reduces calcium availability, promotes secondary hyperparathyroidism, and accelerates bone resorption, thereby contributing to decreased bone mass and impaired skeletal quality. At the cellular level, active vitamin D modulates osteoblasts and their activity, thereby influencing the balance between bone formation and resorption [44]. Beyond skeletal health, vitamin D participates in muscle function and immune regulation, indirectly affecting fall risk and fracture susceptibility [44].

Moreover, micronutrient status may also influence cardiometabolic risk profiles in pediatric cancer survivors. In this context, higher estimated intakes of zinc, copper, and selenium, as well as B vitamins, particularly niacin and riboflavin, have been associated with a lower likelihood of reduced HDLc levels in ALL survivors [45]. Biologically, zinc and selenium contribute to antioxidant defense systems [46,47], copper is involved in redox-active enzymatic pathways [46], niacin influences lipoprotein metabolism [48], and riboflavin serves as a cofactor in mitochondrial oxidative reactions [49]. Collectively, these mechanisms provide a pathophysiological basis linking micronutrient intake with lipid regulation.

Routine high-dose supplementation cannot currently be recommended without biochemical confirmation of deficiency, as evidence supporting generalized supplementation strategies in pediatric oncology remains limited and requires individualized assessment [50,51].

In clinical practice, nutritional care should be individualized according to diagnosis, treatment modality, nutritional risk, and treatment-related symptoms, rather than relying on uniform dietary prescriptions [52]. When spontaneous intake is insufficient, practical strategies include increasing meal energy density, using energy- and protein-rich foods, offering small, frequent meals, and considering oral nutritional supplements when requirements cannot be met through diet alone [52].

### 4.2. Physical Activity and Exercise

Cancer cells exhibit metabolic reprogramming characterized by increased glycolysis and lactate production even under aerobic conditions, a phenomenon known as the Warburg effect. Although these tumor-specific adaptations differ from the metabolic responses induced by exercise, PA improves mitochondrial function, substrate utilization, and systemic metabolic efficiency in host tissues [53]. These physiological adaptations may contribute to improved energy regulation and fatigue tolerance in pediatric oncology patients [53].

Recent data further highlight the clinical relevance of physical inactivity during treatment. Among 133 hospitalized pediatric patients with ALL, 44.4% exhibited low PA levels, while 35.3% showed moderate activity [54]. Chemotherapy exposure, screen time, and social anxiety were significant determinants of reduced activity [54].

Current scientific evidence supports physical exercise as a safe and potentially powerful non-pharmacological intervention in pediatric oncology. Exercise interventions initiated during hospitalization have demonstrated significant short- and medium-term improvements in physical fitness, cardiorespiratory fitness (CRF), and psychosocial well-being in children and adolescents undergoing cancer treatment [55,56,57,58,59]. Initiation from the time of diagnosis is both safe and feasible if sessions are supervised by qualified professionals [57,60,61]. The most frequent reasons for session postponement or cancellation include fever, infection, thrombocytopenia, nausea, bone pain, and severe fatigue [55,56,57,58].

From a clinical implementation perspective, exercise prescription should be guided by individualized functional assessment and delivered in coordination with rehabilitation professionals and the oncology team, considering age, diagnosis, treatment phase, clinical contraindications, and patient preferences [62].

Structured exercise targets the two main pathophysiological axes described in Figure 1. Resistance training may attenuate musculoskeletal deterioration [61], while aerobic exercise improves cardiometabolic risk profiles (adipose–metabolic axis) [53,57]. In addition, regular structured PA may contribute to improved autonomic modulation and fatigue reduction [32]. This integrative perspective reinforces exercise as a central component of multimodal lifestyle interventions in pediatric oncology.

Resistance training protocols are typically structured using moderate repetition ranges (8–15 repetitions), targeting major muscle groups through multi-joint exercises. Early hospital-based programs in children with ALL demonstrated feasibility during active treatment using low-volume approaches (e.g., one set per exercise across 11 exercises) [63]. Subsequent trials have progressively increased training volume to 2–3 sets per exercise, which maintain similar repetition ranges and incorporate rest intervals of 1–2 min [60,64]. These interventions are commonly performed 2–3 times per week and have shown consistent benefits in muscular strength and functional mobility during treatment, with sustained effects reported in survivors [58,61,65]. Observational data further support progressive resistance training in patients with sarcopenic phenotypes [15,66]. Nevertheless, resistance-training protocols remain difficult to compare due to heterogeneity in supervision, design, setting, duration, progression criteria, intensity reporting, and outcome measures across studies [61,67]. This variability should be considered when interpreting the current evidence and translating it into clinical practice.

Aerobic exercise is generally prescribed as continuous moderate-intensity training. Most protocols define intensity within a range of approximately 50–70% of age-predicted maximum heart rate (HRmax), with session durations around 20–30 min [60,63]. While some interventions provide clearly defined intensity thresholds (e.g., 50–80% HRmax) [68], others describe intensity more broadly as “moderate” without standardized quantification [69,70]. The considerable variability in exercise intensity prescription and monitoring across studies limits comparability between interventions and may partially explain the inconsistent cardiorespiratory findings reported in the literature [67].

Importantly, the initiation of structured exercise from the time of diagnosis is both safe and feasible, as it contributes to the preservation of CRF compared with usual care [57]. These findings support the early implementation of exercise rather than delaying it due to concerns about treatment-related toxicity.

Besides traditional resistance–aerobic models, multicomponent interventions that incorporate coordination tasks, adapted sports, and recreational activities have also been implemented. These programs typically maintain moderate intensity (~60–70% HRmax) and are designed to enhance adherence and engagement [71]. Additionally, some interventions combining aerobic exercise with relaxation techniques, as well as virtual reality–based exercise approaches, have reported improvements in sleep quality in pediatric oncology patients [72,73].

Overall, most interventions are mixed or multimodal, combining resistance and aerobic training, and in some cases, psychosocial components [59]. Emerging evidence suggests that even modest increases in moderate-to-vigorous PA may positively influence BMD in pediatric cancer survivors [74]. However, heterogeneity in exercise prescription, particularly regarding intensity and volume, remains a major limitation for standardizing recommendations.

## 5. Multimodal Interventions: Combined Exercise and Nutritional Approaches

The main characteristics and findings of the included studies are summarized in Table 1.

### 5.1. Effects on Physical Function and Body Composition

Interventions that included a structured exercise component showed the most consistent effects on physical function and fitness outcomes. A home-based exercise and nutrition program in children with ALL during maintenance therapy resulted in increased PA levels and improved CRF compared with controls, although nutrient intake and anthropometric measures were similar between groups [75]. Similarly, an 8-week exercise and nutrition education program in adolescent and young adult cancer survivors improved CRF, flexibility, muscular strength, and fatigue-related outcomes, without significant changes in anthropometrics [80].

More targeted exercise programs produced specific adaptations. A 9-month online bone-strengthening exercise intervention did not improve femoral neck areal BMD, the primary outcome, but significantly improved total hip BMC, suggesting that skeletal responses may be specific and dependent on the mechanical characteristics of the intervention [82].

In contrast, interventions based on behavioral counseling or digital health promotion showed less consistent effects on PA levels. No significant changes in objectively measured PA were observed after a 12-week web-based lifestyle intervention, despite improvements in dietary intake and parental feeding practices [77]. Similarly, little evidence of behavior change attributable to a mobile health application was reported, although feasibility, retention, and user satisfaction were encouraging [78].

Taken together, these findings suggest that improvements in physical function are most likely when the exercise component is structured, progressive, and sufficiently dosed. However, the translation of these improvements into broader changes in body composition remains inconsistent.

### 5.2. Effects on Nutritional, Dietary, and Metabolic Outcomes

Across the included interventions, changes were generally more evident in dietary behaviors than in nutritional status or metabolic outcomes. Increased intake of milk, calcium, and protein, alongside reduced potato consumption and lower parental “pressure to eat”, failed to produce significant changes in BMI, waist circumference, or PA levels [77]. Specific dietary improvements were observed after structured interventions [81]. Total caloric intake decreased while calcium intake increased in the intervention group. Participants with higher involvement in the nutritional component also showed greater protein-derived energy intake compared with controls. In contrast, cardiometabolic outcomes did not show statistically significant improvements, although a positive trend toward improved cardiometabolic health was observed among adolescents in the intervention group [81].

The strongest evidence of a metabolic effect comes from a trial where combining caloric and nutrient restriction with exercise during induction therapy failed to reduce fat mass gain in the overall cohort [79]. However, this intervention did reduce fat gain in overweight or obese participants and was associated with a lower risk of minimal residual disease. The intervention also increased adiponectin and reduced insulin resistance, suggesting that lifestyle modulation during treatment may influence metabolic physiology and possibly treatment response [79].

Other interventions showed weaker nutritional effects. No significant differences in energy or nutrient intake were observed between intervention and control groups during longer home-based programs [75]. Improvements in nutrition knowledge, including healthy food choices and label reading, were reported without significant changes in dietary intake [80]. A tailored weight management intervention based on calorie reduction, food logs, portion control, healthier food choices, and PA goals showed benefits in weight maintenance that were more evident in older participants. However, no significant changes in serum metabolic parameters, such as HbA1c, fasting glucose, triglycerides, cholesterol, or blood pressure were observed [76].

Therefore, although nutrition education and behavioral counseling can improve knowledge and selected dietary behaviors, their impact on measurable nutritional status, body composition, or metabolic outcomes seems to be limited unless the intervention includes a more structured, individualized, and monitored nutritional program. Importantly, metabolic effects may occur even when conventional anthropometric changes are modest, emphasizing the value of biochemical and body composition monitoring beyond BMI alone.

### 5.3. Feasibility and Limitations

Exercise–nutrition interventions were shown to be feasible across different phases of the cancer trajectory. A home-based exercise and nutrition program was successfully implemented during maintenance therapy in children with acute lymphoblastic leukemia [75], while other interventions were conducted in adolescent and young adult survivors after treatment completion [80]. Moreover, combined lifestyle interventions have also been applied during intensive treatment phases, such as induction therapy, supporting their feasibility even in difficult clinical contexts [79]. No adverse events were reported across the included studies, even in those involving impact-based exercise such as jumping activities [82].

Despite these encouraging feasibility findings, several methodological limitations must be considered when interpreting the results. Many studies were pilot or feasibility trials with small sample sizes, limiting statistical power and generalizability [75,77,78,80]. Some studies lacked a randomized control group or used historical or convenience controls, which limits causal interpretation [79,81].

In addition, there was substantial heterogeneity in intervention programs, duration, intensity, treatment phase, cancer type, and outcome assessment [78,81,82]. This variability complicates comparisons across studies and limits the ability to define optimal intervention characteristics. Another key limitation is that most studies evaluated isolated domains rather than integrated physiological pathways. The studies focused mainly on fitness [75,80], diet [77,81], or weight management and metabolic markers [76,79]. The fragmentation observed presents challenges in discerning whether exercise and nutrition interact synergistically or simply produce parallel effects.

Overall, while multimodal interventions are feasible and generally well accepted, their translation into robust and sustained physiological or clinical improvements remains limited. This highlights the need for adequately powered randomized trials, standardized intervention protocols, longer follow-up periods, and multidimensional outcome assessment to better understand the effectiveness and mechanisms of combined exercise–nutrition interventions in pediatric oncology.

## 6. Monitoring Health Status During Interventions

Regular monitoring is crucial in pediatric oncology as cancer and its treatment can cause rapid changes in nutritional status and body composition. Single-time evaluations may not capture these changes effectively, underscoring the importance of periodic reevaluation [83]. Therefore, longitudinal monitoring of anthropometric, biochemical, dietary, and functional indicators may help detect early changes and guide timely decisions on nutritional care, rehabilitation referral, and exercise adaptation according to clinical status and treatment phase [52,62].

Body composition and bone health are primarily assessed using dual-energy X-ray absorptiometry (DXA), which represents the reference method for evaluating BMD and lean mass [84]. DXA enables evaluation of total body, lumbar spine (L1–L4), total hip, and femoral neck BMD, as well as lean mass indices such as appendicular lean mass index (ALMI) [66]. Importantly, combining structural and functional criteria allows a more accurate characterization of treatment-related muscle alterations.

In hospital environments, more accessible tools such as bioelectrical impedance analysis (BIA) and ultrasonography are often used to assess body composition [85,86]. BIA represents a non-invasive and feasible method that provides estimates of fat-free mass, skeletal muscle mass, and body water compartments based on resistance and reactance measurements [86]. BIA-derived phase angle has been described as an indicator of body cell mass and cellular integrity and has shown prognostic value in clinical populations [87]. Alterations in muscle mass represent a key component of sarcopenia, whose diagnosis requires not only the assessment of muscle quantity but also muscle strength and physical performance [86].

Concurrently, muscle ultrasound is becoming more popular as a convenient method for assessing both muscle mass and quality. In pediatric oncology, ultrasound-derived parameters such as muscle thickness, cross-sectional area, and intramuscular adipose tissue have shown significant associations with appendicular skeletal muscle mass, muscle strength, and functional performance [85].

Furthermore, biochemical assessment constitutes a fundamental component of nutritional evaluation in pediatric oncology, complementing anthropometric, clinical, and dietary data [88]. It is essential to assess micronutrient status through biochemical methods, as dietary intake does not necessarily reflect circulating concentrations [89]. Blood biomarkers provide relevant information on protein status, organ function, bone health, anemia, and micronutrient deficiencies. In addition, laboratory parameters such as C-reactive protein allow the assessment of inflammatory status, which may also influence the interpretation of nutritional biomarkers [88].

CRF is most commonly assessed using cardiopulmonary exercise testing (CPET), which remains the gold standard for evaluating peak oxygen uptake (VO_2_peak) [90,91]. However, its application may be limited in clinically unstable or immunocompromised patients. In these contexts, the 6-Minute Walk Test (6MWT) provides a practical and validated submaximal alternative suitable for both hospitalized patients and survivors [90,91,92].

Muscle strength is typically assessed using handgrip or isokinetic dynamometry due to their feasibility and validity in pediatric populations [90]. Functional performance is frequently evaluated using tests such as the Timed Up and Go, Timed Up and Down Stairs, and Sit to Stand, which provide clinically meaningful information on mobility and functional capacity [90,93]. Commonly applied assessment techniques for flexibility include goniometry and the sit-and-reach test to measure range of motion. In addition, balance assessment, often using the Sensory Organization Test or force platforms, allows detection of neuromotor impairments related to neuropathy, corticosteroid exposure, or inactivity [90].

Assessment of habitual PA remains challenging. While self-reported questionnaires are widely used, they are susceptible to recall bias and social desirability effects. Consequently, objective monitoring through accelerometry is increasingly adopted. Devices such as ActiGraph and wearable trackers have demonstrated feasibility in pediatric oncology populations [66,94], although the lack of standardized cut-off thresholds for intensity classification remains a key limitation [95].

Overall, although a wide range of assessment tools is available, the lack of standardized outcome measures remains a major limitation in the field. Future research should prioritize harmonized protocols integrating CRF, muscular strength, body composition, and objectively measured PA to strengthen the evidence and improve clinical applicability (Table 2).

## 7. Conclusions

Multimodal lifestyle interventions that combine structured exercise and nutrition programs represent potential non-pharmacologic strategies to enhance recovery and mitigate the long-term effects of disease and treatment in pediatric cancer.

Nutritional management strategies should focus on optimizing body composition by ensuring sufficient protein intake and correcting any micronutrient deficiencies. Due to the fluctuating energy needs during treatment, personalized nutritional plans must be regularly reviewed and adjusted.

PA plays a key role in targeting musculoskeletal and metabolic changes. Resistance training helps maintain lean mass, bone mineral content and functional abilities, while aerobic exercise enhances CRF and metabolic function. Supervised exercise programs can be safely introduced early in the treatment process. Sleep patterns may also influence autonomic regulation, highlighting the importance of a holistic approach.

Metabolic dysregulation in pediatric oncology is influenced by a complex interplay of inflammatory pathways, neuroendocrine signals, and treatment-related factors, causing appetite changes, energy imbalances, and metabolic issues. However, the clinical relevance of these changes remains unclear, and future studies should integrate metabolic and autonomic outcomes to better understand underlying mechanisms.

Despite favorable results, heterogeneity in intervention design and outcome assessment remains a major limitation. Utilizing validated tools to assess body composition, physical fitness, muscle strength, and biochemical markers enables a more precise evaluation of patient status and facilitates tailored intervention planning. Harmonizing intervention protocols and assessment frameworks in future research can enhance evidence quality and facilitate practical implementation in pediatric oncology settings.

## Figures and Tables

**Table 1:** Characteristics and Main Findings of Multimodal Exercise and Nutritional Interventions in Pediatric Oncology.

Study	Design	Population	Intervention	Findings	Limitations/Bias
Moyer-Mileur et al., 2009 [75]	RCT	ALL Maintenance phase Intervention (*n* = 6) Control (*n* = 7)	12 months Home-based 3 sessions/week (MVPA intensity) Nutritional education	Increased PA Improved CRF No differences in nutritional status	Small sample size
Huang et al., 2014 [76]	Pilot RCT	ALL Survivor Intervention (*n* = 19) Control (*n* = 19)	4 months ≥1 h MVPA/day Caloric restriction Increased consumption of healthy foods	≥14 years old: weight maintained/↑ PA ↓ Self-reported negative mood	Incomplete accelerometer and dietary data
Zhang et al., 2019 [77]	Pilot quasi-experimental	ALL Maintenance phase/≤2 years post-treatment *n* = 15	12 weeks Nutrition education PA promotion (≥60 min/day; bone-strengthening ≥3 days/week) Parent-focused behavioral intervention	↑ Milk, calcium and protein intake ↓ Potatoes consumption No significant changes in PA and BMI	Small sample size Single-arm design
Fuemmeler et al., 2020 [78]	Pilot quasi-experimental	ALL/Lymphoma Survivors *n* = 15	8 weeks App-based gamified intervention promoting PA and healthy diet education	↓ MVPA App users maintained PA levels ↑ Fruit and vegetable self-efficacy	Small sample size Single-arm design
Orgel et al., 2021 [79]	nRCT	ALL Induction phase Intervention (*n* = 40) Control (*n* = 80)	4 weeks Home-based Daily PA (aerobic + resistance) Daily caloric deficit (≥10%)	↓ Fat gain in OW/OB ↓ MRD risk Improved insulin sensitivity	Non-randomized design Historical control Short duration Low adherence
DeNysschen et al., 2021 [80]	Pilot quasi-experimental	Pediatric cancer Survivors *n* = 24	8 weeks 1-h supervised session/week (aerobic + resistance) 3-home-based sessions/week Nutritional education	↑ CRF ↑ Muscular strength ↑ QoL ↑ Nutrition knowledge	Small sample size
Delorme et al., 2025 [81]	Non-randomized controlled study	Pediatric cancer Intervention (*n* = 45) Control (*n* = 77)	12 months Supervised 2–3 sessions/week 45 min MVPA (aerobic + resistance) Individualized nutrition	↓ Energy intake ↑ Calcium intake Favorable BP and HbA1c trends	Non-randomized design Convenience sample Historical control
Marmol-Perez et al., 2026 [82]	Assessor-blinded RCT	Pediatric cancer Survivors Intervention (*n* = 58) Control (*n* = 58)	9 months Video-recorded 3–4 days/week, 3–4 sets, 10–20 reps (jumps/squats) Calcium/vitamin D recommendations	↑ Total hip BMC z-score No adverse events	Unsupervised home-based sessions

Notes: ALL, acute lymphoblastic leukemia; BMC, bone mineral content; BMI, body mass index; BP, blood pressure; CRF, cardiorespiratory fitness; HbA1c, glycated hemoglobin; MRD, minimal residual disease; MVPA, moderate-to-vigorous physical activity; OW/OB, overweight/obese; PA, physical activity; QoL, quality of life; RCT, randomized controlled trial; nRCT, non-randomized controlled trial; ↑ indicates an increase; ↓ indicates a decrease.

**Table 2:** Summary of tools and techniques for assessment.

Tool/Technique	Measured Variable
Dual-energy X-ray absorptiometry	Bone mineral density; Lean mass; Body composition
Bioelectrical impedance analysis	Body composition; Phase angle
Muscle ultrasound	Muscle thickness; Muscle quality
Blood biomarkers	Micronutrient status; Protein status; Inflammation; Anemia; Organ function
Cardiopulmonary exercise test	Peak oxygen uptake; Cardiorespiratory fitness
6-Min Walk Test	Cardiorespiratory fitness
Handgrip/Isokinetic dynamometry	Muscle strength
Functional tests (Timed Up and Go, Sit-to-Stand)	Motor function
Goniometry/Sit and reach test)	Flexibility; range of motion
Force platform	Postural control; Neuromuscular function
Accelerometry	Physical activity levels

## Data Availability

No new data were created or analyzed in this study. Data sharing is not applicable to this article.

## Abbreviations

The following abbreviations are used in this manuscript:

PA	Physical activity
CRF	Cardiorespiratory fitness
ALL	Acute lymphoblastic leukemia
BMD	Bone mineral density
BMI	Body mass index
HDLc	High-density lipoprotein cholesterol
REE	Resting energy expenditure
KD	Ketogenic diet
HRmax	Maximum heart rate
TNF-α	Tumor necrosis factor alpha
IL-1	Interleukin 1
BIA	Bioelectrical impedance analysis
6MWT	6-minute walk test
DXA	Dual-energy X-ray absorptiometry
